# The Adaptation of MCF-7 Breast Cancer Spheroids to the Chemotherapeutic Doxorubicin: The Dynamic Role of Phase I Drug Metabolizing Enzymes

**DOI:** 10.3390/metabo15020136

**Published:** 2025-02-18

**Authors:** Daniel Crispim, Carolina Ramos, Francisco Esteves, Michel Kranendonk

**Affiliations:** 1 Comprehensive Health Research Centre (CHRC) NOVA Medical School | Faculty of Medical Sciences, Universidade NOVA de Lisboa, 1169-056 Lisboa, Portugal; daniel.crispim@nms.unl.pt (D.C.); carolina.s.ramos@nms.unl.pt (C.R.); francisco.esteves@nms.unl.pt (F.E.); 2 Instituto Politécnico de Setúbal (IPS), Escola Superior de Saúde (ESS), Departamento de Ciências Biomédicas, Estefanilha, 2910-761 Setúbal, Portugal

**Keywords:** breast cancer (BC), drug-metabolizing enzymes (DMEs), 3D models/spheroids, doxorubicin (DOX), cytochrome P450s (CYPs), oxidoreductases

## Abstract

**Background/Objectives:** Drug resistance (DR) is a major challenge in cancer therapy, contributing to approximately 90% of cancer-related deaths. While alterations in drug metabolism are known to be key drivers of DR, their role—particularly in the early stages of acquired chemoresistance—remains understudied. Phase I drug-metabolizing enzymes (DMEs), especially cytochrome P450s (CYPs), significantly influence the metabolic fate of chemotherapeutic agents, directly affecting drug response. This study aimed to investigate the role of Phase I DMEs in the early metabolic adaptation of breast cancer (BC) MCF-7 cells to doxorubicin (DOX). **Methods:** Four types of spheroids were generated from MCF-7 cells that were either DOX-sensitive (DOX^S^) or adapted to low concentrations of the chemotherapeutic agent (DOX^A^ 25, 35, and 45 nM). The expression levels of 92 Phase I DMEs and the activities of specific CYP isoforms were assessed in both DOX^S^ and DOX^A^ spheroids. **Results:** A total of twenty-four DMEs, including fifteen CYPs and nine oxidoreductases, were found to be differentially expressed in DOX^A^ spheroids. Pathway analysis identified key roles for the differentially expressed DMEs in physiologically relevant pathways, including the metabolism of drugs, arachidonic acid, retinoic acid, and vitamin D. **Conclusions:** The deconvolution of these pathways highlights a highly dynamic process driving early-stage DOX resistance, with a prominent role of CYP3A-dependent metabolism in DOX adaptation. Our findings provide valuable insights into the underlying molecular mechanisms driving the early adaptation of MCF-7 cells to DOX exposure.

## 1. Introduction

Breast cancer (BC) is the most prevalent malignancy in women, with more than two million new cases and six hundred thousand deaths reported globally in 2022 [1]. Among the multiple strategies available for the treatment of BC patients, including the surgical excision of tumors, hormonal interventions, radiation, and immunotherapy, chemotherapy serves as the cornerstone in advanced BC treatment [2,3]. One of the main contributing factors in treatment failure is drug resistance (DR), a phenomenon causally related to disease progression, relapse, metastasis, and ultimately death [4,5]. Studies have highlighted that a substantial proportion of BC patients develop chemoresistance during treatment, contributing to 90% of all cancer-related fatalities [6]. Intriguingly, despite the well-established connection between DR and treatment failure, the molecular mechanisms driving cancer’s adaptation to drug exposure remain poorly understood, leaving a knowledge gap in the field of oncobiology and therapeutic development.

Doxorubicin (DOX), an anthracycline antibiotic, is one of the most utilized chemo-therapeutic agents in BC treatment [7]. Administered intravenously, typically in combination with other adjuvant chemotherapeutic agents, DOX reaches a maximum plasma concentration of approximately 7 µM [8]. Its anticancer action stems from its ability to inhibit topoisomerase II and generate free radicals, causing DNA damage, cell cycle arrest, membrane disruption, and protein oxidation, ultimately inducing apoptosis. Despite its significant therapeutic efficacy, DOX’s clinical utility is considered limited due to its high cardiotoxicity [9]. DOX metabolism is described to be primarily mediated by cytochrome P450 (CYP) enzymes, including CYP3A4, 2D6, 2B6, and 1B1, while several oxidoreductases such as CYP-oxidoreductase (CPR) may contribute through secondary detoxification pathways [10,11,12].

The development of DR is a multifactorial process involving several mechanisms, including increased drug efflux, enhanced DNA repair, inhibition of apoptosis, immune evasion, drug-target mutations, and altered drug metabolism [13,14]. Among these, drug efflux transporters correspond to the most extensively studied mechanism in DR development [15]. In drug-resistant cancer cells, the upregulation of efflux proteins can rapidly clear chemotherapeutic agents, reducing their intracellular concentrations and therapeutic efficacy. Notably, mRNA expression levels of efflux transporters seem to vary over time as resistance to chemotherapeutics such as imatinib, dasatinib, or DOX progresses [15,16]. These data highlight the complexity of this adaptive mechanism, with different transporters active at various stages of chemoresistance, and suggests that other mechanisms may simultaneously contribute to DR. Although drug efflux has received significant attention in the development of chemoresistance, the role of other mechanisms, including drug metabolism modulation, remains underexplored [17,18,19].

Despite the liver being the primary site of xenobiotic metabolism, containing the highest concentrations of drug-metabolizing enzymes (DMEs), variations in enzyme expression within tumor cells have been shown to significantly impact drug efficacy [20,21,22,23]. Changes in DME activity within cancer cells can accelerate the detoxification and elimination of chemotherapeutic agents, thereby diminishing their efficacy and consequently contributing to chemoresistance. Among DMEs, cytochrome P450 (CYP) enzymes and various oxidoreductases play essential roles in metabolizing both xenobiotics, including drugs and carcinogens, and endobiotics like fatty acids, sterols, and vitamins—molecules integral to the altered metabolic state of cancer cells [23,24,25,26]. CYP enzymes, particularly microsomal isoforms from families 1–3, are predominant in Phase I drug metabolism, accounting for more than 80% of the metabolism of all clinically marketed drugs and approximately 70% of chemical carcinogens [23]. Additionally, numerous studies have linked deviations in the expression and activity of these enzymes to tumorigenesis, cancer progression, and prognosis [27,28,29,30].

Despite their recognized importance in oncobiology, the role of Phase I metabolism enzymes in the development of DR, including the dynamics of their expression and activity throughout this process, remains poorly characterized [31]. This is especially relevant during the initial cellular adaptation to anticancer compounds at subtherapeutic stages of chemoresistance, in which early metabolic changes may trigger DR at therapeutic doses. Insights into these early adaptations will enhance our understanding of the mechanisms underlying the acquisition of DR in cancer. Additionally, elucidating these dynamics may potentially lead to the identification of biomarkers that can be used to predict tumors with a high probability of developing DR at therapeutic levels. Such biomarkers could subsequently be used to develop more patient-tailored chemotherapeutic strategies, ultimately improving treatment decision-making and reducing the burden of cancer.

Given the need to investigate the early adaptive mechanisms leading to DR, our study aimed to explore the role of Phase I DMEs in the primary stages of DOX adaptation in MCF-7 cells. For this purpose, four different types of cells derived from this cell line (luminal A) were applied, representing the most frequent type of BC. A spheroid culture approach was employed to accurately mimic the in vivo tumor microenvironment, offering a more physiologically relevant cell system than conventional monolayer cultures. This 3D method has been demonstrated to be of value in pharmacokinetic and drug resistance studies [32,33,34]. Expression profiles of 92 genes were evaluated, including key CYP isoforms and Phase I oxidoreductases, in which spheroids were either sensitive (DOX^S^) or adapted to withstand low doses of DOX (DOX^A^ 25, 35, and 45 nM). Additionally, we measured relevant CYP enzyme activities to verify whether altered transcription levels indeed translated to functional metabolic alterations.

## 2. Materials and Methods

### 2.1. Reagents

Dulbecco’s modified Eagle’s medium—low glucose (DMEM), fetal bovine serum (FBS), penicillin-streptomycin (10,000 units penicillin and 10 mg streptomycin per mL), insulin, dimethyl sulfoxide (DMSO), trypsin, cytochrome *c* (cyt *c*) (horse heart), glucose-6-phosphate, glucose-6-phosphate dehydrogenase, nicotinamide-adenine dinucleotide phosphate (NADPH), ethoxyresorufin (EthR), coumarin (Coum), phosphate-buffered saline pH 7.4 (PBS), dibenzylfluorescein (DBF), dimethyl sulfoxide (DMSO), 3-cyano-7-ethoxycoumarin (CEC), acetonitrile (ACN), TRIzol Reagent, and sodium dithionite were obtained from Merck (Darmstadt, Germany). DOX, nicotinamide adenine dinucleotide phosphate (NADP^+^), collagenase Type IV, rodent collagen I, and trypan blue, were obtained from Fisher Scientific (Waltham, MA, USA). Seakem LE agarose was obtained from Lonza Bioscience (Walkersville, MD, USA). TaqMan Universal Master Mix II (no UNG) was obtained from Applied Biosystems (Foster City, CA, USA). Zombie violet fixable viability dye was obtained from BioLegend (San Diego, CA, USA). Insulin was obtained from Cell Applications, Inc. (San Diego, CA, USA). Bradford reagent was obtained from Bio-Rad (Hercules, CA, USA). Quiazol was obtained from Qiagen (Hilden, Germany). All other chemicals and solvents were of the highest grade commercially available.

### 2.2. MCF-7 Monolayer (2D) Cultures

The MCF-7 cell line was purchased from DSMZ-German Collections of Microorganisms and Cell Culture GmbH (Braunschweig, Germany) (MCF-7, ACC 115). MCF-7 cells were cultured in growth medium (DMEM with 10% FBS, 1% penicillin-streptomycin, and 10 µg/mL insulin). Culture medium was replaced every 2–3 days and cells were sub-cultured by trypsinization when confluency reached approximately 80%. Cells were incubated in a 5% CO_2_ humidified chamber at 37 °C. To promote the adaptation to low DOX concentrations (25, 35, or 45 nM), MCF-7 cells underwent stepwise exposures to increasing doses of the chemotherapeutic agent. DOX^A^ cells were cultured in the presence of DOX every third medium change, to maintain their adaptation level. Spheroids derived from parental MCF-7 cells (DOX^S^) were cultured in parallel and used as controls.

### 2.3. DOX Toxicity Assay Using MTT

MCF-7 DOX^S^ and DOX^A^ cells were seeded in 96-well plates at a density of 1 × 10^4^ cells per well and cultured for 48 h, as described above. Following this incubation, 100 µL of fresh medium containing varying concentrations of DOX (0, 100, 200, 400, 800, 1500, 3000, or 6000 nM) was added to each well and incubated for an additional 48 h. MTT was then added to each well at a final concentration of 0.5 mg/mL and incubated for 3 h. The resulting formazan crystals were dissolved in 100 µL of DMSO, and absorbance was measured at 570 nm using a Zenyth 3100 microplate reader (Anthos, Wals-Siezenheim, Austria). Cell viability was calculated as a percentage relative to the untreated control.

### 2.4. MCF-7 Spheroid (3D) Cultures

MCF-7 spheroids were developed by applying the microwell liquid overlay technique, as previously described [35]. Initially, DOX^A^ cells were cultured in monolayer format and exposed to DOX at concentrations matching their adaptation levels for 24 h. Subsequently, cells obtained from these 2D cultures were resuspended at the indicated cell density, and growth medium was supplemented with collagen at a final concentration of 10 µg/mL. A cell suspension (200 µL) was added to each agarose-coated well of a 96-well plate, followed by centrifugation at 1000× *g* for 10 min at room temperature and incubation at 37 °C with 5% CO_2_. Every three days, half of the medium was replaced with fresh medium. Between days nine and ten of spheroid development, DOX^A^ spheroids were re-exposed to their respective DOX concentrations to maintain DR-related gene expression. As a control, DOX^S^ cells were exposed to the same concentration of the DMSO solvent used to prepare the DOX solutions. All spheroids were collected on day ten of development for subsequent studies. Spheroid images were captured using a Zeiss Axiovert 40 CFL microscope (Carl Zeiss AG, Oberkochen, Germany) at a total magnification of 50×, and their diameters, circularity, and compactness were assessed using AnaSP software (version 2.0).

### 2.5. Cell Viability

The viability of both 2D- and 3D-cultured MCF-7 cells was analyzed using Zombie Violet dye. After the trypsinization of the 2D cultures, suspensions containing 1 × 10^5^ cells were centrifuged at 500× *g* for 5 min at 4 °C, and the cell pellets were washed using 1 mL of PBS. After a second centrifugation, the pellets were resuspended in 100 µL of a Zombie Violet solution (1:1000 in PBS) and incubated for 15 min in a light-protected chamber. Subsequently, excess dye was removed by performing two washing steps with 500 µL of PBS supplemented with 2% FBS, and the cell pellets were resuspended in 200 µL of this solution. Cell suspensions were then analyzed using the FACSCanto II flow cytometer (BD Biosciences, Franklin Lakes, NJ, USA). For the viability assessment of 3D-cultured cells, five spheroids with a starting seeding number of approximately 2 × 10^4^ cells each were pooled. After a PBS wash, spheroids were dissociated into single cells by incubation with collagenase (Type IV, 100 units/mL) for 45 min, followed by a 5 min trypsin incubation. Subsequent steps followed the protocol for monolayer-cultured cells. All viability measurements were performed in triplicate. Negative controls were included, prepared similarly but without Zombie Violet incubation.

### 2.6. Expression Levels of CYPs and Oxidoreductases in DOX^A^ Spheroids

#### 2.6.1. RNA Isolation and cDNA Synthesis

Two hundred and forty spheroids of each of the four levels of DOX^A^ were collected, washed with cold PBS, and resuspended in 800 µL of TRIzol Reagent. RNA extractions were subsequently carried out according to the Direct-zol^TM^ RNA Miniprep Plus Kit’s protocol (Zymo Research, Irvine, CA, USA). Total RNA from these spheroids was used to generate cDNA using the High-Capacity RNA-to-cDNA^TM^ Kit (Applied Biosystems, Waltham, MA, USA). In short, 1.7 µg of total RNA was used per 20 µL reaction, following the manufacturer’s instructions. The generated cDNA was quantified using a Nanodrop 2000 Spectrophotometer (Thermo Scientific, Waltham, MA, USA) and stored at −20 °C for subsequent use [36].

#### 2.6.2. RT-qPCR

The expression of 92 Phase I enzymes was assessed using TaqMan^TM^ Array “Human CYP450 and other Oxygenases” 96-Well Plates (Applied Biosystems, Waltham, MA, USA). Reactions were prepared following the manufacturer’s guidelines, employing a total cDNA content of 47.2 ng and a final reaction volume of 10 µL per well. Real-time PCR was performed on a QuantStudio^TM^ 5 Real-Time PCR System (Applied Biosystems, Waltham, MA, USA), with thermal cycling conditions set at 95 °C for 10 min, followed by 40 cycles at 95 °C for 15 s, and 60 °C for 1 min. For this analysis, the fold change and respective *p*-values obtained for each target gene were used to determine the differential mRNA expression scores in the MCF-7/DOX^A^ versus the MCF-7/DOX^S^ spheroids. Data analysis was performed as previously described [36]. Each sample was analyzed in duplicate.

### 2.7. CYP Enzyme Activities in DOX^A^ Cell-Derived Microsomes

#### 2.7.1. Subcellular Fraction (Microsomes) Isolation and Characterization

The microsomal fractions of spheroids were obtained using the Mem-PER Plus Membrane Protein Extraction Kit (Thermo Scientific, Waltham, MA, USA). Three hundred spheroids of each of the four types of MCF-7 spheroids were collected, treated with collagenase (see above), and subsequently processed as previously described [36]. The total membrane protein content of these spheroid samples was determined using the Bradford method. Total CYP and CPR quantifications were performed by applying the CO differential spectrophotometric method and the cyt *c* reduction assay, respectively, as previously demonstrated [36].

#### 2.7.2. CYP Activity Assays

The activities of specific CYP isoforms were evaluated by incubating microsomal fractions with four standard CYP probe substrates, as previously detailed [36]. This evaluation entailed the following reactions: ethoxyresorufin-O-de-ethylation (EROD), coumarin 7-hydroxylation (C7H), dibenzylfluorescein-O-debenzylation (DBODF), and cyano-ethoxycoumarin-O-de-ethylation (CECOD). Briefly, all assays were conducted in a 96-well format using a final MCF-7 microsomal protein concentration of 0.2 mg/mL per well. Reactions were performed in triplicate in 100 mM potassium phosphate buffer (pH 7.6) supplemented with 10 mM MgCl_2_ and a NADPH regenerating system (NADP^+^ 200 µM, glucose 6-phosphate 500 µM, and glucose 6-phosphate dehydrogenase 40 mU/mL, final concentrations). The substrate concentrations used were defined as either two (DBF, Coum) or four times (EthR, CEC) their CYP substrates’ K_M_ values, namely 7.5, 5, 20, and 200 µM, respectively. Final solvent concentrations in the reaction mixtures were as follows: 0.1% ACN for DBF and Coum, 0.2% DMSO for EthR, and 0.3% DMSO for CEC. Product formation was observed for 10 min at 37 °C with a multi-mode microtiter plate reader (SpectraMax^®^i3x, Molecular Devices, San José, CA, USA). Rates were calculated using a standard curve of the products.

### 2.8. Statistical Analysis

Results obtained from the RT-qPCR data were statistically analyzed by a mixed-effects model (REML) with Dunnett’s multiple comparisons test. Genes were considered as differentially expressed when *p*-value < 0.05 and fold change was either > 2 or <0.5. Differences in CYP and CPR contents, viability, and CYP kinetic activity assays were analyzed using the unpaired t-test. All statistical analyses were conducted using the GraphPad Prism software (version 8.0.2).

## 3. Results

### 3.1. DOX Toxicity Assay

**Figure 1:**
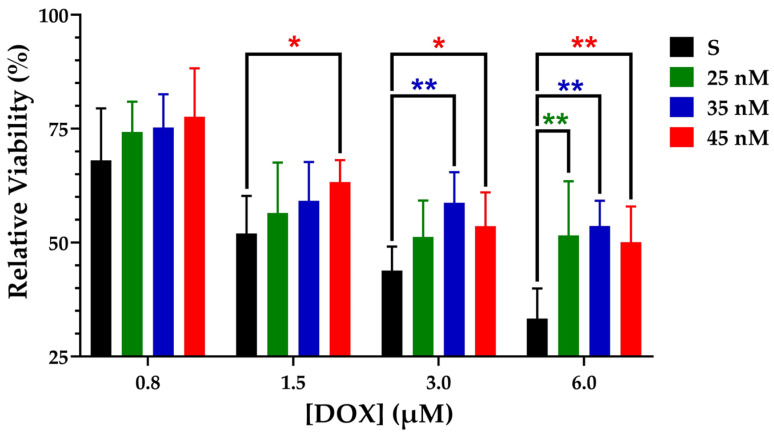
Normalized cell viability of DOX^S^ and DOX^A^ cells treated with varying concentrations of DOX. Values are represented as the mean ± SD (N = 10) (* *p* < 0.01, ** *p* < 0.001).

To confirm the higher resistance of developed MCF-7/DOX^A^ cells to DOX exposure, an MTT assay was conducted. Cells were exposed to DOX concentrations up to 6000 nM (see Figure 1 and Supplemental Figure S1). Relative viability values for each level of DOX adaptation are provided in Supplemental Tables S1–S4.

These results demonstrate the significantly higher viability of DOX^A^ 45 nM cells compared to DOX^S^ cells when exposed to 1.5 µM of DOX. At 3 µM, both DOX^A^ 35 nM and 45 nM cells exhibited significantly higher viability. Furthermore, when exposed to 6 µM, DOX^A^ 25, 35, and 45 nM cells all showed a significant increase in viability.

### 3.2. MCF-7 Spheroid Development and Characterization

**Figure 2:**
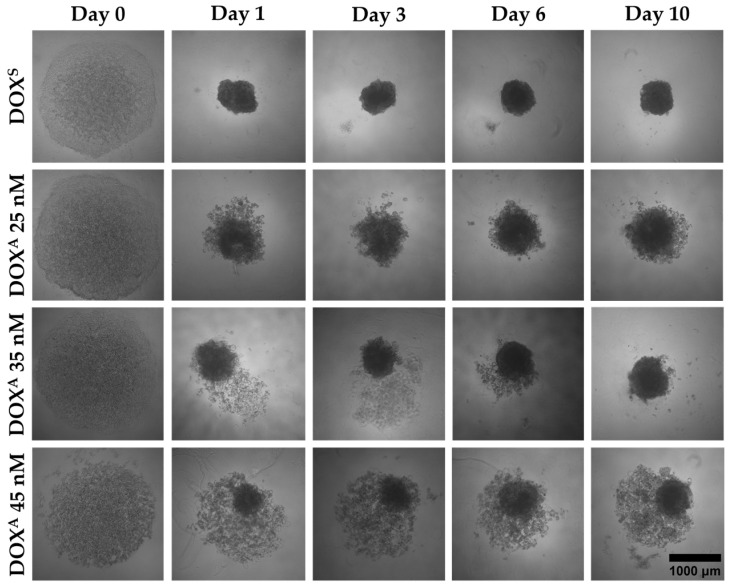
Representative brightfield images of spheroids derived from the four types of MCF-7 cells (DOX^S^ and DOX^A^ 25, 35, and 45 nM) over a 10-day culture period.

To study the impact of Phase I DME expression and activity on the development of early DOX adaptation, a 3D cell model was set up to mimic the microenvironment of malignant cells [37]. An initial cell seeding density of 2 × 10^4^ cells/spheroid was chosen, ensuring sufficient cellular material for subsequent analyses [38,39]. The procedure included centrifugation (1000× *g*, 10 min), creating a uniform cell distribution at the bottom of the non-adherent well surfaces on day 0, and the addition of collagen type I to culture medium to promote cellular aggregation [40,41]. Subsequently, this method was applied to all four types of MCF-7 cells (DOX^S^ and DOX^A^ 25, 35, and 45 nM cells) (Figure 2).

**Figure 3:**
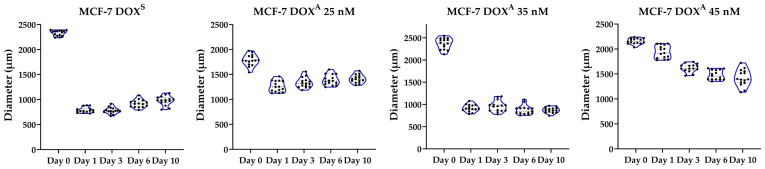
Diameter analysis of the four analyzed types of MCF-7 spheroids over a 10-day culture period (N = 15).

Characterization of the spheroids was performed following the MISpheroID Consortium guidelines [42]. This characterization was conducted by assessing the respective diameters at multiple time points (days 0, 1, 3, 6, and 10) (Figure 3). Data on the circularity and compactness of the constructs are shown in Supplemental Figure S2. MCF-7 spheroids exhibiting distinct levels of DOX adaptation demonstrated different morphologies. By day ten, most DOX^S^ and DOX^A^ 35 nM cells had aggregated into single, spheroid structures. However, a significant fraction of DOX^A^ 25 nM and particularly 45 nM cells failed to fully aggregate, resulting in larger and less compact aggregates. Overall, diameters exhibited minimal variation within levels of DOX adaptation, demonstrating the reproducibility of the formation procedure.

The development of a necrotic core in spheroids is a very common phenomenon in 3D cultures due to the limited nutrient and oxygen diffusion to the center of these structures [43]. To address this concern, cell viability was assessed on days zero and ten of the spheroid formation procedure. A similar and statistically significant viability decline across all four types of MCF-7 cells was observed, as depicted in Table 1, reflecting that this decline is independent of the cells’ DOX adaptation level.

### 3.3. Gene Expression Profiles of CYPs and Oxidoreductases in MCF-7 Spheroids

The expression profiles of 92 CYP enzyme complex protein factors and oxidoreductases were analyzed in the four types of MCF-7 spheroids to assess potential expression changes throughout early DOX adaptation. A list of the RT-qPCR data of all analyzed genes is presented in Supplemental Table S5.

**Figure 4:**
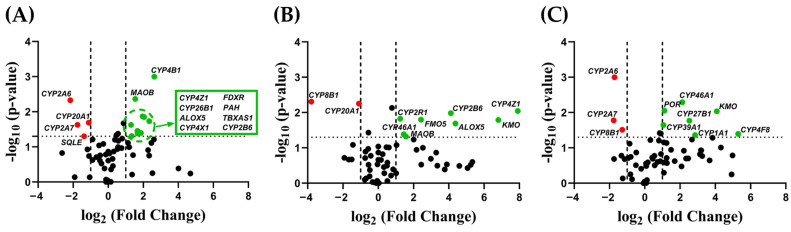
Differential gene expression of 92 DMEs in MCF-7/DOX^A^ spheroids relative to parental MCF-7/DOX^S^ spheroids ((**A**) MCF-7/DOX^A^ 25 nM, (**B**) MCF-7/DOX^A^ 35 nM, (**C**) MCF-7/DOX^A^ 45 nM). Genes with a fold change > 2 (upregulated, shown in green) or <0.5 (downregulated, shown in red) and a *p*-value < 0.05 were considered significantly differentially expressed (N = 2).

Among the ninety-two evaluated target genes, twenty-four were found to be differentially expressed in the different DOX^A^ spheroids, comprising a total of fifteen CYPs and nine oxidoreductases. Notably, out of the twenty-four differentially expressed enzymes, five were downregulated, while the remaining nineteen were found to be upregulated (Figure 4). Several genes exhibited simultaneous upregulated expression in both DOX^A^ 25 and 35 nM spheroids (i.e., *CYP2B6*, *CYP4Z1*, *ALOX5*, and *MAOB*), while others were found to be concurrently upregulated in 35 and 45 nM adapted spheroids (i.e., *CYP27B1* and *KMO*). Interestingly, none of the examined genes was detected as simultaneously differentially expressed across the three levels of DOX^A^ spheroids, suggesting a highly dynamic adaptative process in the early stages of DOX resistance acquisition. The transcript levels of certain genes showed particularly high levels of upregulation in adapted cells (fold change > 15), particularly for DOX^A^ 35 nM and 45 nM cells, namely *CYP2B6*, *ALOX5*, *CYP4Z1*, *KMO,* and *CYP4F8*, respectively (Supplemental Figure S3).

Several of the analyzed genes exhibited no amplification in any of the four studied types of MCF-7 spheroids, which may be the result of very low (i.e., below the detection threshold) or nonexistent levels of these genes’ transcripts. These included the CYP isoforms of the CYP11 family (*CYP11A1* and *CYP11B1/2*), as well as *CYP2C18*, *CYP2C19*, *CYP2A13*, *CYP2F1*, *CYP7A1*, *CYP3A4*, and several oxidoreductases, namely tryptophan hydroxylases 1 and 2 (*TPH1/2*) and flavin-containing monooxygenases 1, 2, and 3 (*FMO1/2/3*).

### 3.4. CYP/CPR Protein Levels and Activities of MCF-7 Spheroids

Gene transcription levels do not always directly correlate with their protein levels or enzymatic activities [44]. To investigate this relationship, we isolated and characterized the microsomal fractions from each of the four types of MCF-7 spheroid cells. These fractions were subjected to various assays to determine whether DOX adaptation correlates with changes in protein level and/or enzyme activities. Microsomal samples primarily contain CYP enzymes, which are critical for Phase I drug metabolism, along with CPR, their obligatory electron donor [27,45,46].

Total CYP content in the microsomal fractions of DOX^A^ 35 and 45 nM spheroids showed a significant increase when compared to the DOX^S^ spheroids (Table 2). Regarding CPR quantification, only DOX^A^ 35 nM spheroids displayed a statistically significant change, with reduced protein levels compared to parental cells. This aligns with the RT-qPCR results, which showed approximately a threefold decrease in the CPR gene (*POR*) transcripts in the 35 nM spheroids, although this reduction was not statistically significant. In contrast, even though *POR* expression was upregulated in DOX^A^ 45 nM cells, no corresponding increase in CPR protein content was observed for this DOX adaptation level.

**Figure 5:**
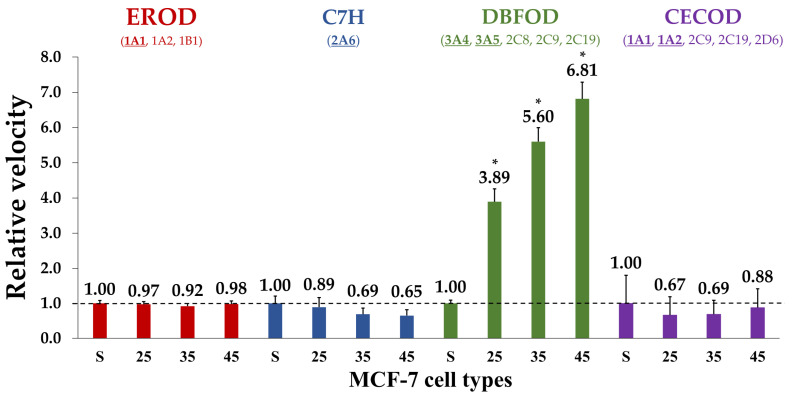
Normalized relative velocities of CYP activities in MCF-7 spheroids [MCF-7/DOX^A^/MCF-7/DOX^S^, FU/min]. FU = Fluorescent units. Values are represented as mean ± SD (N = 3) (* *p* < 0.001) (underlined CYPs: main isoform(s) involved).

The activity profiles of specific microsomal CYP isoforms were analyzed by conducting multiple enzyme kinetic assays to confirm potential deviations in drug metabolism during DOX adaptation (Figure 5). The activity of the three major CYP subfamilies involved in drug metabolism were verified, namely CYP1A, 2A and 3A. CYP1A-mediated metabolism, assessed via EROD activity, remained stable throughout the development of early DOX adaptation. These results were further supported by the observed CECOD activity levels (also specific for isoforms of the CYP1A family), showing no significant variation across all MCF-7 cell types. CYP2A6-mediated C7H activity showed a tendency to decrease throughout the early development of DOX adaptation, although these changes were not statistically significant. In contrast, CYP3A-dependent DBFOD activity displayed a statistically significant increase across all analyzed levels of DOX^A^ cells.

## 4. Discussion

This study aimed to elucidate the role of 92 genes, including key CYP isoforms and Phase I oxidoreductases, in the early stages of DOX adaptation, using spheroid cultured MCF-7 cells as an in vitro BC model.

The MCF-7/DOX^A^ cells exhibited increased resistance to DOX at therapeutic concentrations (1–7 µM), as demonstrated by the MTT viability assay. This finding supports our hypothesis that early metabolic alterations associated with chemoresistance are relevant in the development of DR at clinically relevant doses.

Regarding MCF-7 spheroid morphology, studies have shown that different cell types require tailored methodologies for spheroid formation [35,37]. This suggests that the adapted MCF-7 cells seem to resemble different cell types, where exposure to different DOX concentrations may have altered the expression of cell–cell adhesion and matrix proteins.

**Figure 6:**
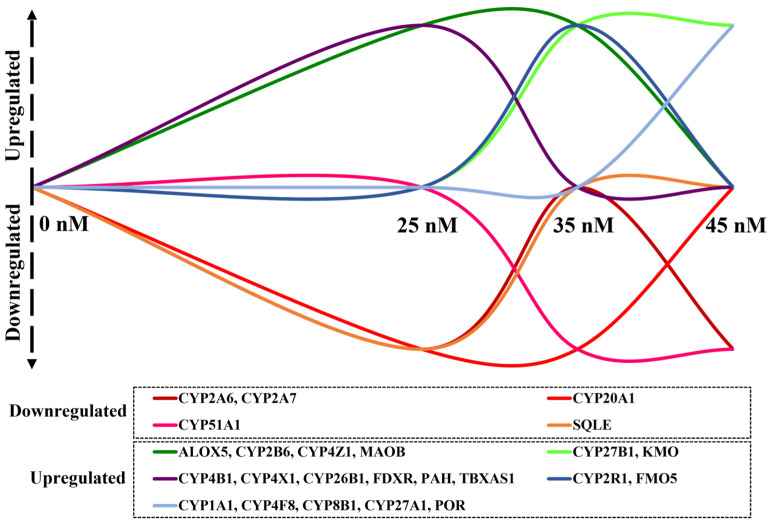
Dynamics of the expression of the 24 differentially expressed genes detected in MCF-7/DOX^A^ spheroids at three levels of DOX adaptation.

Analysis of DME expression in MCF-7 spheroids revealed several enzymes to be altered during early adaptation to DOX. Similarly to the alterations observed in the expression of drug efflux transporters during chemotherapeutic treatment [15], our findings underscore the dynamic nature of early chemoresistance development and its associated mechanisms, as demonstrated in Figure 6.

In total, twenty-four genes were differentially expressed in DOX^A^ spheroids, with five showing significant upregulation: *CYP2B6*, *ALOX5*, *CYP4Z1*, and *KMO* increased by 17-, 20-, 112-, and 240-fold, respectively, in DOX^A^ 35 nM spheroids, and *CYP4F8* by 39-fold in DOX^A^ 45 nM spheroids. *CYP2B6* expression has been associated with elevated BC risk, with pharmacogenetic studies emphasizing its influence on drug metabolism [47,48]. ALOX enzymes are involved in xenobiotic and drug metabolism, with *ALOX5* overexpression linked to cell metastasis in ovarian cancer [49]. *CYP4Z1*, which showed the highest overexpression, has previously been linked to tamoxifen resistance and increased tumor growth in BC [29,50]. *KMO* has been correlated with poor prognosis and accelerated breast tumor progression [51]. While *CYP4F8*’s role in tumor progression is well established, its correlation with DR remains unclear [52]. The observed variability in gene expression among MCF-7 spheroids with different degrees of DOX adaptation may be the result of epigenetic modifications, copy number alterations, or changes in transcriptional regulation linked to metabolic reprogramming in response to DOX exposure [53,54].

Pathway analysis of the twenty-four differentially expressed genes in DOX^A^ MCF-7 spheroids suggests that four physiological processes were impacted during early adaptation to DOX. Upregulation of genes associated with drug metabolism and DR, including *CYP1A1*, *CYP2B6*, *CYP4Z1*, *CYP26B1*, *CYP51A1*, *FMO5*, and *POR*, points to an enhanced metabolic capacity within DOX^A^ spheroids. This suggests an accelerated biotransformation of DOX, reducing its cytotoxic effects and promoting cell survival. Additionally, *CYP1A1*, a key Phase I DME, has previously been linked to increased breast cancer risk, while high *CYP26B1* has been associated with poor prognosis in colorectal cancer [55,56,57]. *POR* is crucial for xenobiotic and steroid metabolism [23]. Its upregulation in DOX^A^ spheroids enhances CYP activity, supporting an adaptive response that diminishes DOX’s therapeutic efficacy [58].

Five genes involved in the arachidonic acid metabolic pathway—*CYP4Z1*, *CYP4F8*, *ALOX5*, *TBXAS1*, and *POR*—were significantly overexpressed in DOX^A^ spheroids. Notably, arachidonic acid metabolism and subsequent eicosanoid synthesis have previously been linked to the release of growth factors, angiogenic factors, and pro-inflammatory regulators [59,60,61]. This may indirectly regulate inflammatory responses, which in turn can modulate DME expression [62].

Two genes involved in retinoic acid metabolism, *CYP26B1* and *CYP2A6*, were notably overexpressed in DOX^A^ spheroids. Retinoic acid acts as a signaling molecule with inhibitory effects on carcinogenesis, cell growth, and cancer cell survival [63]. Overexpression of these enzymes in DOX^A^ cells indicates an adaptive response by accelerating retinoic acid metabolism, reducing its inhibitory impact on cellular expansion.

Two genes involved in vitamin D biosynthesis, *CYP2R1* and *CYP27B1*, were upregulated in DOX^A^ spheroids. Vitamin D deficiency has previously been linked to poorer cancer treatment outcomes and the reversal of drug resistance [64,65,66]. This antitumor effect contrasts with our findings, as two enzymes responsible for the formation of the biologically active form of vitamin D were overexpressed in DOX^A^ cells. Nevertheless, the overexpression of these enzymes may indicate an adaptive response involving vitamin D metabolism during early DOX treatment, warranting further investigation.

**Figure 7:**
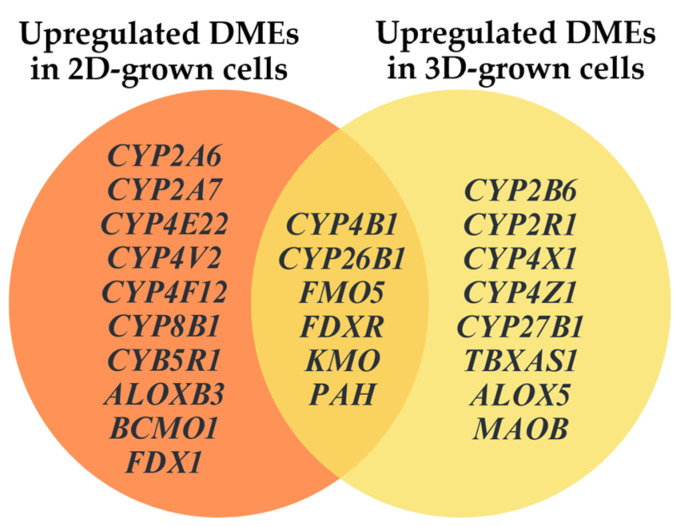
Venn diagram illustrating the similarities and discrepancies in DME expression profiles between 2D- and 3D-cultured MCF-7/DOX^A^ 25 and 35 nM cells.

By comparing these results with our previous study analyzing DOX^A^ 25 and 35 nM MCF-7 cells cultured in a monolayer, we identified a set of gene transcripts—*CYP4B1*, *CYP26B1*, *FDXR*, *FMO5*, *KMO*, and *PAH*—to be upregulated in cells cultured in both the 2D and 3D models (Figure 7) [36]. This overlap suggests that this specific group of enzymes may significantly contribute to subtherapeutic resistance to DOX in MCF-7 cells. Notably, several differences in DME expression profiles were still observed between the 2D and 3D setups (see also Supplemental Tables S6 and S7), highlighting the significant influence of cellular spatial organization on enzyme expression, as previously described [67,68].

The CPR/CYP ratios observed in the DOX^S^ and DOX^A^ 25 nM microsomal fractions were higher than those typically reported for human liver microsomes, which range from 1:5 to 1:15 [69,70]. In contrast, spheroids adapted to higher DOX concentrations (DOX^A^ 35 and 45 nM) showed a significant increase in total CYP content. These findings support the hypothesis that these cells have adapted to higher DOX concentrations by increasing their CYP-mediated detoxification capacity [11,45,69,70].

Enzymatic activity measurements of specific CYP isoforms confirmed the altered CYP-dependent metabolism of DOX^A^ MCF-7 cells. EROD activity remained consistent across all types of MCF-7 spheroids, despite the elevated *CYP1A1* transcript levels detected in DOX^A^ 45 nM cells. CYP2A6-mediated C7H activity showed no significant variation, although its observed decrease correlated with reduced *CYP2A6* transcripts in DOX^A^ 25 and 45 nM cells. DBFOD activity, primarily catalyzed by CYP3A4/5, was significantly increased in all DOX^A^ spheroids. Interestingly, even though CYP3A4 is recognized as an important DOX metabolizer, *CYP3A4* expression was not detected in any of the performed RT-qPCR analyses [71]. In contrast, the observed five-, twenty-four-, and ninefold increases in *CYP3A5* expression in DOX^A^ spheroids seem to explain the enhanced DBFOD activity. Augmented activity of CYP3A-dependent metabolism in a DOX-resistant status has also previously been reported [36,72,73].

Considerable discrepancies were observed among gene transcript levels, protein contents, and enzymatic activities, confirming that protein and activity phenotypes do not necessarily mirror transcriptional responses [36,44]. Considering prior studies on this topic, an intricate relationship may stem from a range of regulatory mechanisms, including CYP polymorphisms, transcriptional co-factors, mRNA stability, post-translational modifications, catalytic turnover, and protein–protein interactions within the metabolic network [23,36,46,74,75].

## 5. Conclusions

Our findings indicate that variations in DME expression and activity contribute to the development of subtherapeutic DOX adaptation in MCF-7 cells, suggesting complex, dynamic, and pathway-specific mechanisms in the development of low-level chemoresistance. Enzymes involved in both xenobiotic and endobiotic metabolism were found to be differentially expressed at various stages of early DOX adaptation, leaving clues that chemoresistance likely arises from a coordinated network of altered expressions and enzymatic activities.

The existence of such DR networks in BC in general requires further investigation using different cell lines to represent the different molecular subtypes of BC, as well as more-complex multicell-type spheroid models. These could be accompanied by time-spatial functional characterization to elucidate the underlying regulatory mechanisms driving these changes at transcriptional and post-translational levels. Moreover, epigenetic alterations associated with acquired DOX adaptation warrant further exploration, as these may provide additional insights into the adaptability of resistant phenotypes.

Additionally, studies using patient-derived samples could help determine whether targeting metabolic reprogramming, particularly CYP3A-dependent metabolism, could reverse or prevent DOX resistance. Such findings could contribute to the development of potential therapeutic strategies aimed at overcoming chemoresistance in BC patients.

## Figures and Tables

**Table 1:** Cell viability on days 0 and 10 of MCF-7 spheroid formation.

MCF-7 Cells	Live Cells (%)	
	Day 0	Day 10
DOX^S^	94.5 ± 0.6	76.6 ± 2.5 *
DOX^A^ 25 nM	94.2 ± 2.7	69.5 ± 3.0 *
DOX^A^ 35 nM	94.7 ± 1.9	68.6 ± 0.3 *
DOX^A^ 45 nM	96.1 ± 1.5	73.0 ± 4.6 *

Values are represented as the mean ± SD (N = 3) (* *p* < 0.0001).

**Table 2:** Total CYP and CPR contents in microsomal fractions of MCF-7/DOX^S^ and DOX^A^ cells.

Microsomal Fraction	Contents		
	CPR	CYP	CPR/CYP
	(pmol/mg Protein) ^1^		Ratios
MCF-7/DOX^S^	44.5 ± 4.4	142.7 ± 34.7	1:3
MCF-7/DOX^A^ 25 nM	47.0 ± 5.2	84.1 ± 19.2	1:2
MCF-7/DOX^A^ 35 nM	25.0 ± 0.2 *	370.9 ± 32.2 *	1:15
MCF-7/DOX^A^ 45 nM	37.0 ± 2.3	257.8 ± 22.6 *	1:7

^1^ CYP (N = 3) and CPR (N = 2) values are represented as the mean ± SD (* *p* < 0.01).

## Data Availability

The original contributions presented in this study are included in the article/Supplementary Material. Further inquiries can be directed to the corresponding author.

## Supplementary Materials

The following supporting information can be downloaded at: https://www.mdpi.com/article/10.3390/metabo15020136/s1, Figure S1: Normalized cell viability of DOX^S^ and DOX^A^ cells treated with varying concentrations of DOX (0, 100, 200, 400, 800, 1500, 3000, and 6000 nM concentrations plotted on a logarithmic scale, N = 10) (* *p* < 0.01, ** *p* < 0.001). Figure S2: Circularity and compactness analyses of the four analyzed types of MCF-7 spheroids over a 10-day culture period; Figure S3: Fold changes of differentially expressed genes in MCF-7/DOX^A^ spheroids, relative to the parental DOX^S^ spheroids. Table S1: Relative viability of MCF-7/DOX^S^ cells 48 h after DOX exposure. Table S2: Relative viability of MCF-7/DOX^A^ 25 nM cells 48 h after DOX exposure. Table S3: Relative viability of MCF-7/DOX^A^ 35 nM cells 48 h after DOX exposure. Table S4: Relative viability of MCF-7/DOX^A^ 45 nM cells 48 h after DOX exposure. Table S5: C_t_ values obtained for each target gene of the RT-qPCR assays; Table S6: Upregulated genes detected in 2D- and 3D-cultured MCF-7 DOX^A^ cells. Table S7: Downregulated genes detected in 2D- and 3D-cultured MCF-7 DOX^A^ cells.

## Abbreviations

The following abbreviations are used in this manuscript:

ACN	Acetonitrile
BC	Breast cancer
C7H	Coumarin 7-hydroxylation
CEC	3-Cyano-7-ethoxycoumarin
Coum	Coumarin
CECOD	Cyano-ethoxycoumarin-O-de-ethylation
CPR	Cytochrome P450-oxidoreductase
CYP	Cytochrome P450
Cyt *c*	Cytochrome *c*
DBF	Dibenzylfluorescein
DBODF	Dibenzylfluorescein-o-debenzylation
DMEM	Dulbecco’s modified Eagle’s medium
DME	Drug-metabolizing enzyme
DOX	Doxorubicin
DMSO	Dimethyl sulfoxide
DOX^A^	DOX-adapted
DOX^S^	DOX-sensitive
DR	Drug resistance
EROD	Ethoxyresorufin-O-de-ethylation
EthR	Ethoxyresorufin
