# Supplementary material for: The Adaptation of MCF-7 Breast Cancer Spheroids to the Chemotherapeutic Doxorubicin: The Dynamic Role of Phase I Drug Metabolizing Enzymes
Source: Metabolites. 2025 Feb 18;15(2):136. doi: 10.3390/metabo15020136 (PMC11857127; doi:10.3390/metabo15020136)
Supplement: Supplementary file 1 [file metabolites-15-00136-s001.zip › metabolites-3462762-supplementary.pdf]

Supplementary Material

The Adaptation of MCF-7 Breast Cancer Spheroids to the Chemotherapeutic Doxorubicin: The Dynamic Role of Phase I Drug Metabolizing Enzymes

Daniel Crispim[1], Carolina Ramos[1], Francisco Esteves[1] and Michel Kranendonk[1]*

[1] Comprehensive Health Research Centre (CHRC) NOVA Medical School | Faculty of Medical Sciences, Universidade NOVA de Lisboa

* Correspondence: michel.kranendonk@nms.unl.pt;

Table S1. Relative viability (%) of MCF-7/DOX^S cells 48 hours after DOX exposure.

DOX (μM)	Relative Viability (%)										Mean
**0**	89.29	104.04	90.64	81.57	101.82	82.81	115.63	79.58	136.08	118.53	**100.00**
**0.1**	96.73	95.02	110.13	101.90	136.54	99.85	104.99	75.88	99.72	72.16	**99.29**
**0.2**	73.01	89.18	103.21	89.42	113.80	95.57	107.33	83.74	111.52	100.20	**96.70**
**0.4**	79.37	86.23	75.73	93.65	121.34	90.55	118.59	72.39	111.97	106.00	**95.58**
**0.8**	48.20	61.36	61.27	62.51	82.73	73.83	78.73	75.07	57.44	79.43	**68.06**
**1.5**	51.54	47.17	63.82	58.29	63.86	48.06	39.98	56.48	44.74	46.07	**52.00**
**3.0**	50.76	41.77	42.66	46.71	46.39	49.99	39.00	47.93	37.54	36.06	**43.88**
**6.0**	22.49	35.64	28.39	43.27	30.23	32.56	40.78	39.34	33.99	26.63	**33.33**

Table S2. Relative viability (%) of MCF-7/DOX^A 25 nM cells 48 hours after DOX exposure.

DOX (μM)	Relative Viability (%)										Mean
**0**	99.61	77.35	99.42	97.71	102.56	93.05	117.79	104.88	88.87	118.74	**100.00**
**0.1**	91.23	75.97	90.01	95.86	97.84	87.44	97.81	109.65	122.40	112.31	**98.05**
**0.2**	91.28	75.76	93.93	86.86	88.99	104.35	103.56	88.44	114.63	127.54	**97.53**
**0.4**	85.64	75.22	81.32	74.30	92.00	93.38	98.84	111.69	102.44	126.28	**94.11**
**0.8**	74.04	77.78	65.57	76.51	64.71	85.47	69.45	81.42	71.36	76.36	**74.27**
**1.5**	56.90	45.33	43.10	74.11	42.39	61.36	49.33	63.84	68.92	59.50	**56.48**
**3.0**	57.42	42.98	40.49	57.89	45.46	47.67	48.30	60.50	63.71	48.05	**51.25**
**6.0**	39.15	48.08	36.15	47.79	60.58	44.29	43.13	70.74	59.41	66.36	**51.57**

Table S3. Relative viability (%) of MCF-7/DOX^A 35 nM cells 48 hours after DOX exposure.

DOX (μM)	Relative Viability (%)										Mean
**0**	105.89	106.10	94.15	81.52	105.11	96.49	102.32	71.51	115.27	121.65	**100.00**
**0.1**	87.61	91.82	96.60	74.79	98.24	98.58	110.64	80.28	113.65	109.03	**96.12**
**0.2**	100.75	88.61	93.15	71.28	105.76	105.15	126.03	86.09	114.51	100.06	**99.14**
**0.4**	93.30	86.12	82.53	96.85	89.79	98.25	105.31	134.40	104.75	108.17	**99.95**
**0.8**	79.38	69.22	81.16	59.24	80.42	78.39	81.02	76.36	79.07	68.58	**75.28**
**1.5**	52.90	51.67	72.98	54.79	63.73	45.72	67.80	66.95	55.59	59.61	**59.18**
**3.0**	59.44	48.34	60.79	61.08	61.67	54.26	67.37	57.63	67.99	48.67	**58.72**
**6.0**	47.86	47.49	57.23	53.72	65.78	58.49	50.69	51.77	50.92	52.41	**53.64**

Table S4. Relative viability (%) of MCF-7/DOX^A 45 nM cells 48 hours after DOX exposure.

DOX (µM)	Relative Viability (%)										Mean
**0**	86.26	92.73	96.16	82.94	92.11	108.41	100.47	82.30	122.25	136.36	**100.00**
**0.1**	94.64	81.52	89.49	95.82	98.55	98.36	98.22	95.16	112.38	112.96	**97.71**
**0.2**	98.69	85.21	84.38	71.53	100.16	90.97	82.24	86.88	123.40	108.41	**93.19**
**0.4**	89.17	89.66	84.26	89.27	93.66	108.42	95.36	92.81	110.20	100.84	**95.36**
**0.8**	73.30	66.45	84.92	62.21	72.64	75.56	91.70	71.19	83.58	94.50	**77.61**
**1.5**	64.16	60.95	71.21	66.88	56.56	64.70	67.17	59.20	65.43	56.85	**63.31**
**3.0**	50.31	42.09	48.31	66.51	57.75	47.46	53.05	55.86	63.33	51.18	**53.58**
**6.0**	46.94	34.14	50.28	53.82	49.29	45.71	53.43	46.07	61.58	59.67	**50.09**

Table S5. C_t values obtained for each target gene of the RT-qPCR assays.

Gene Symbol	MCF-7 Cells							
	DOX^S (1)	DOX^S (2)	DOX^A 25nM (1)	DOX^A 25nM (2)	DOX^A 35nM (1)	DOX^A 35nM (2)	DOX^A 45nM (1)	DOX^A 45nM (2)
**18S**	11.33	10.90	11.03	10.68	26.01	25.64	11.92	11.76
**GAPDH**	16.08	16.02	16.34	16.47	19.68	19.47	16.80	16.75
**HPRT1**	20.93	20.97	22.02	21.98	22.32	22.18	22.49	22.18
**GUSB**	23.15	23.10	23.34	23.49	24.35	24.31	23.90	23.60
**CYP1A1**	26.51	26.38	25.07	26.54	28.39	28.65	24.67	24.33
**CYP1A2**	33.86	33.18	33.68	34.24	U	U	33.88	32.94
**CYP1B1**	22.14	22.12	22.17	22.28	24.70	24.73	22.85	22.60
**CYP11A1**	U	U	U	U	U	U	U	U
**CYP11B1**	U	U	U	U	U	U	U	U
**CYP11B2**	U	U	U	U	U	U	U	U
**CYP17A1**	U	U	U	U	U	U	U	U
**CYP19A1**	U	U	U	U	U	U	U	U
**CYP2A13**	U	U	U	U	U	U	U	U
**CYP2A6**	29.86	29.82	32.58	32.56	34.56	32.88	32.60	32.35
**CYP2A7**	29.41	29.32	31.66	31.70	33.84	32.27	32.20	31.89
**CYP2B6**	30.96	31.16	30.22	30.43	29.03	28.88	28.30	27.96
**CYP2C18**	U	U	U	U	U	U	U	U
**CYP2C19**	U	U	U	U	U	U	U	U
**CYP2C8**	34.09	33.60	33.95	35.43	33.96	32.99	34.84	34.45
**CYP2C9**	U	U	U	U	U	U	U	U
**CYP2D6**	32.70	31.93	U	U	U	U	35.72	34.42
**CYP2E1**	30.13	30.36	33.79	33.16	33.50	31.98	32.65	32.32
**CYP2F1**	U	U	U	U	U	U	U	U
**CYP2J2**	27.07	24.72	27.51	27.52	28.66	28.68	27.76	27.31
**CYP2R1**	26.12	26.08	26.94	26.94	26.95	26.78	27.19	26.88
**CYP2S1**	32.67	31.98	34.42	33.68	36.88	35.80	32.50	32.23
**CYP2U1**	27.26	27.04	28.46	28.77	29.39	29.02	29.65	28.50
**CYP2W1**	33.70	33.28	34.61	34.00	U	U	34.98	35.58
**CYP3A4**	U	U	U	U	U	U	U	U
**CYP3A43**	U	U	U	U	U	U	U	U
**CYP3A5**	34.02	33.31	31.85	32.06	31.74	30.59	31.81	31.12

Table S5. (Cont.)

**CYP3A7**	U	U	U	U	U	U	U	U
**CYP4A11, CYP4A22**	U	U	U	U	U	U	U	U
**CYP4B1**	29.68	29.89	27.65	27.77	29.43	29.10	27.77	27.27
**CYP4F11**	27.02	26.97	26.98	27.01	28.66	28.72	27.18	27.03
**CYP4F12**	32.43	22.65	30.08	29.97	30.72	29.86	29.94	29.48
**CYP4F2**	33.16	33.45	33.18	33.41	U	U	33.66	32.71
**CYP4F22**	32.04	31.92	31.25	31.45	33.88	32.81	32.03	31.30
**CYP4F3**	U	U	U	U	U	U	U	U
**CYP4F8**	35.69	34.61	33.08	33.13	33.87	32.82	30.95	30.59
**CYP4V2**	28.08	28.16	28.94	29.36	30.00	29.58	29.48	29.57
**CYP4X1**	34.55	35.06	33.55	33.86	32.16	31.13	32.78	32.17
**CYP4Z1**	33.79	33.86	32.30	32.37	27.98	27.87	30.12	29.44
**CYP7A1**	35.62	35.99	U	U	32.95	32.09	34.44	34.58
**CYP7B1**	U	U	U	U	U	U	U	U
**CYP8B1**	32.70	32.39	30.54	30.75	31.81	30.75	31.04	30.82
**CYP20A1**	25.21	25.21	26.78	26.98	28.36	28.27	27.21	26.64
**CYP21A2**	U	U	U	U	U	U	U	U
**CYP24A1**	31.19	30.99	31.98	32.09	34.24	32.70	33.09	33.22
**CYP26A1**	30.26	30.03	30.68	30.43	31.76	30.94	29.11	28.37
**CYP26B1**	29.42	28.94	27.43	27.37	32.92	30.97	29.24	28.51
**CYP26C1**	U	U	U	U	U	U	U	U
**CYP27A1**	33.40	33.38	34.43	34.85	35.87	34.26	33.38	33.13
**CYP27B1**	28.49	28.41	28.17	28.34	29.06	28.98	27.35	27.11
**CYP27C1**	U	U	U	U	U	U	U	U
**CYP39A1**	U	U	U	U	U	U	U	U
**CYP46A1**	31.29	31.24	31.34	31.36	33.94	33.73	32.18	31.85
**CYP51A1**	22.03	22.01	23.12	23.09	27.82	27.84	24.26	24.17
**TBXAS1**	35.00	35.29	33.65	33.84	34.19	32.84	32.82	32.01
**PTGIS**	U	U	U	U	U	U	U	U
**POR**	22.85	22.72	22.15	22.30	26.24	26.22	22.71	22.45
**CYB5A**	31.60	31.35	33.22	33.22	34.83	32.88	33.19	31.91
**CYB5B**	23.07	23.16	24.17	24.13	25.94	25.76	24.38	23.57
**CYB5R1**	24.95	24.50	23.75	23.98	26.31	26.15	24.18	23.06
**CYB5R2**	31.21	31.01	29.19	29.11	30.50	29.82	29.94	29.21
**CYB5R3**	23.87	23.75	24.01	24.19	26.29	26.20	23.97	23.65
**CYB5R4**	26.03	26.05	26.24	26.46	27.13	26.89	26.48	25.99

Table S5. (Cont.)

**FDX1**	**30.88**	**30.89**	**31.34**	**31.84**	**32.46**	**31.44**	**32.01**	**32.01**
**FDXR**	25.94	25.73	25.01	25.10	28.62	28.56	25.51	25.02
**FLAD1**	25.10	25.00	25.72	25.77	26.50	26.41	25.75	25.33
**NENF**	23.95	23.92	24.10	24.13	25.24	25.11	24.34	24.27
**PGRMC1**	22.19	30.93	22.40	22.47	23.52	23.49	22.80	22.30
**PGRMC2**	22.88	22.68	23.24	23.38	24.42	24.24	23.80	23.45
**BCMO1**	U	U	U	U	U	U	U	U
**DBH**	U	U	U	U	U	U	U	U
**DOHH**	30.66	30.53	30.68	30.58	33.54	32.71	30.46	30.08
**FMO1**	U	U	U	U	U	U	U	U
**FMO2**	U	U	U	U	U	U	U	U
**FMO3**	U	U	U	U	U	U	U	U
**FMO4**	27.52	27.45	28.16	28.04	28.94	29.02	28.47	28.16
**FMO5**	26.83	26.83	26.59	26.61	26.50	26.33	26.82	26.50
**KMO**	31.91	31.53	32.03	31.90	26.96	26.88	28.65	28.44
**PAH**	30.02	29.97	28.89	28.88	28.62	28.84	28.42	28.05
**PAM**	25.02	26.60	24.03	24.05	25.66	25.66	25.10	24.95
**SC4MOL**	24.74	24.64	26.10	26.20	27.17	27.36	26.08	26.16
**SQLE**	22.34	22.25	24.16	24.32	24.21	24.09	24.14	23.99
**TH**	U	U	U	U	U	U	U	U
**TPH1**	U	U	U	U	U	U	U	U
**TPH2**	U	U	U	U	U	U	U	U
**TYR**	U	U	U	U	U	U	U	U
**MAOA**	27.19	27.12	27.61	27.68	28.96	28.87	27.79	27.24
**MAOB**	27.53	27.45	26.48	26.55	27.96	27.90	28.17	27.75
**ALOXE3**	27.12	26.85	26.72	26.63	28.98	28.94	27.21	26.90
**ALOX5**	29.86	29.83	28.46	28.62	27.52	27.44	27.82	27.30
**ALOX12**	U	U	U	U	U	U	U	U
**ALOX15**	30.14	35.06	29.44	28.93	35.21	34.25	29.02	29.44

*U: no amplification detected.

Table S6. Upregulated genes detected in 2D- and 3D-cultured MCF-7 DOX^A cells.

Upregulated Genes					
MCF-7 DOX^A 25 nM			MCF-7 DOX^A 35 nM		
**2D**	**Shared**	**3D**	**2D**	**Shared**	**3D**
CYP2A6, CYP4F12, CYP8B1, ***KMO***	***CYP26B1, PAH***	*CYP2B6, CYP4Z1, CYP4X1,* ***CYP4B1****, ALOX5,* ***FDXR****, MAOB, TBXAS1*	*CYP2A7,* ***CYP4B1****, CYP4F12, CYP4F22, CYP4V2, CYP8B1,* ***CYP26B1****, CYB5R1, FDX1,* ***FDXR****, BCMO1,* ***PAH****, ALOXE3*	*FMO5,* ***KMO***	*CYP2B6, CYP4Z1, CYP2R1, CYP27B1, ALOX5, MAOB*

*Genes detected in both analyses but in different levels of DOX^A are represented in bold.

Table S7. Downregulated genes detected in 2D- and 3D-cultured MCF-7 DOX^A cells.

Downregulated Genes			
MCF-7 DOX^A 25 nM		MCF-7 DOX^A 35 nM	
**2D**	**3D**	**2D**	**3D**
CYP2D6, CYP2S1	CYP2A6, CYP2A7, CYP20A1, SQLE	CYP3A5	CYP20A1, CYP51A1

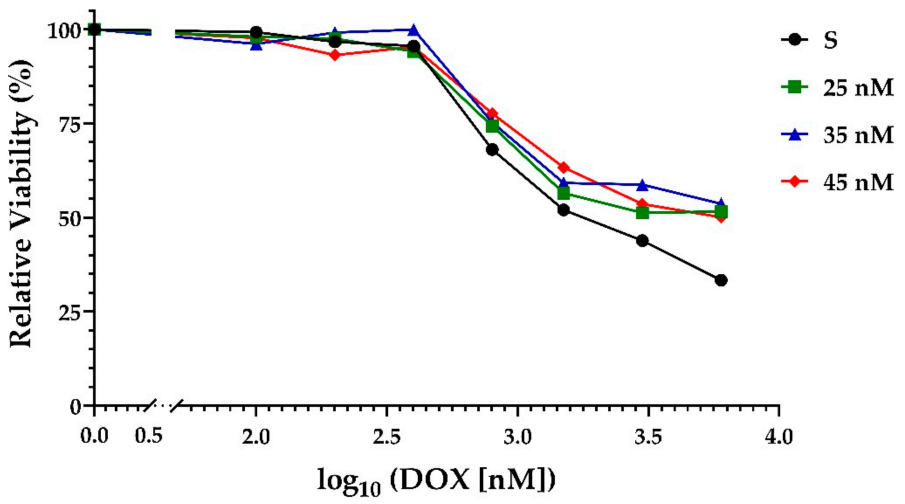

Figure S1. Normalized cell viability of DOX^S and DOX^A cells treated with varying concentrations of DOX (0, 100, 200, 400, 800, 1500, 3000, and 6000 nM concentrations plotted on a logarithmic scale, N = 10) (* P < 0.01, ** P < 0.001).

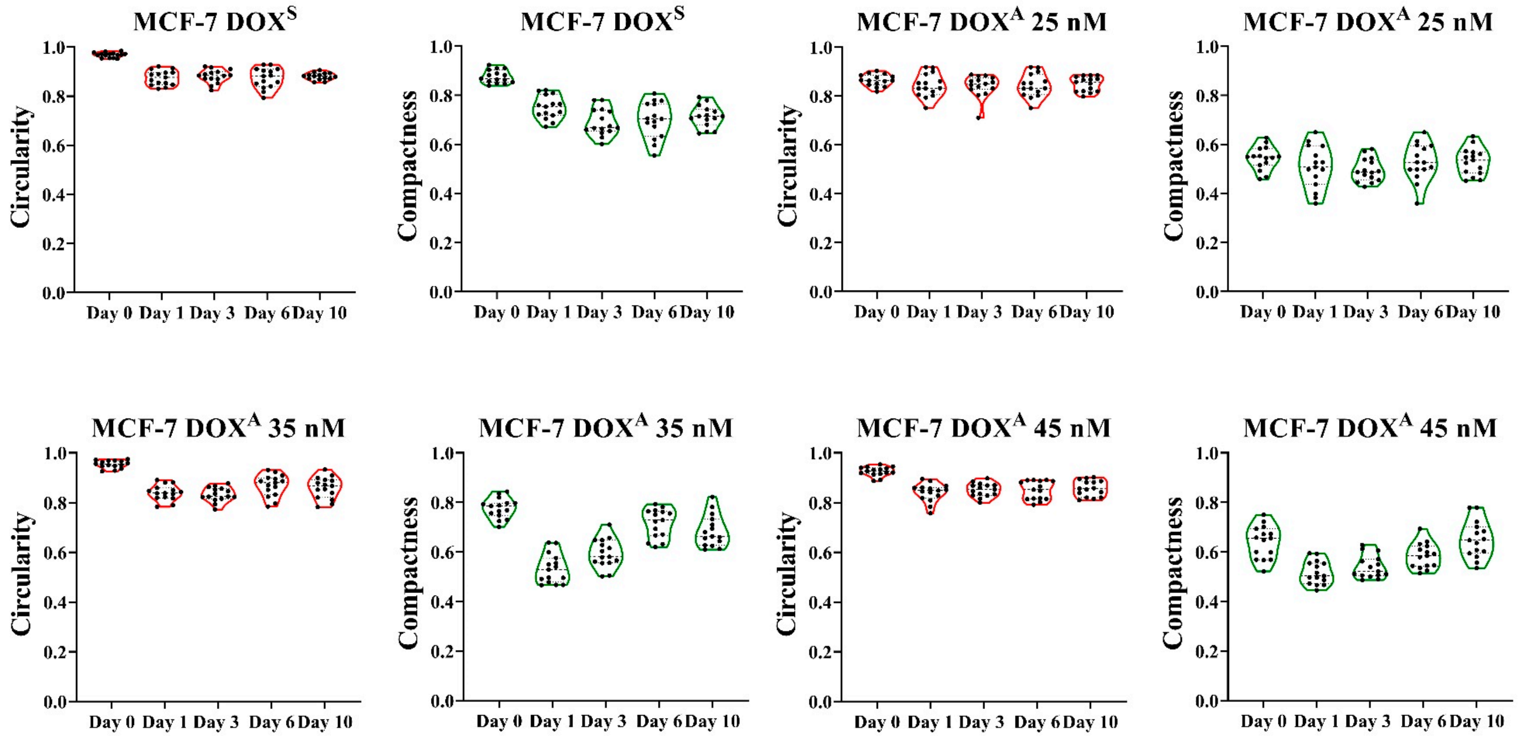

Figure S2. Circularity and compactness analyses of the four types of MCF-7 spheroids over a 10-day culture period. Both parameters were assessed using the AnaSP software (N = 15).

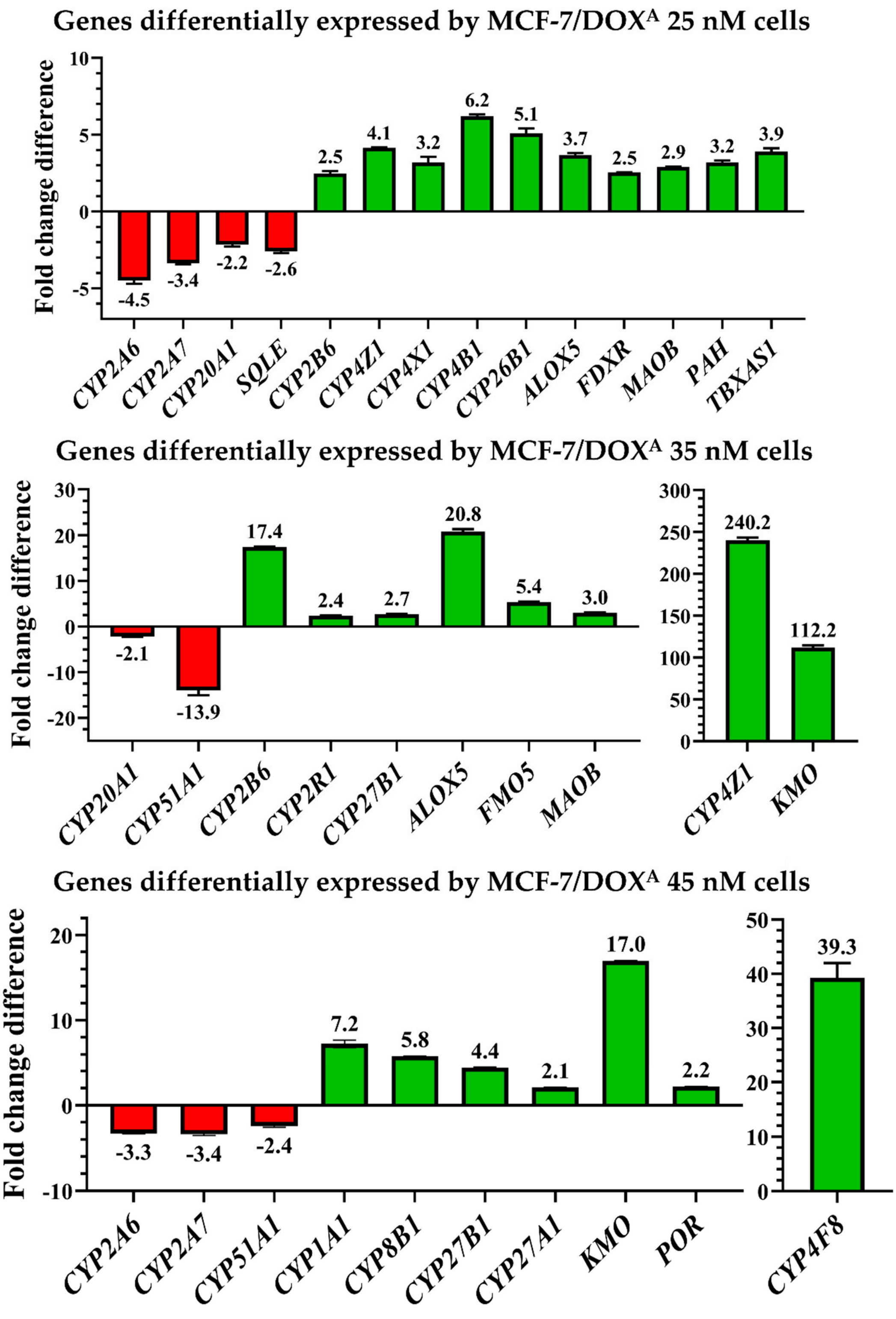

Figure S3. Fold changes of differentially expressed genes in MCF-7/DOX^{A} spheroids, relative to the parental DOX^{S} spheroids (*CYP27A1*: Sterol 27-hydroxylase; *CYP27B1*: 1α-hydroxylase; *CYP51A1*: Lanosterol 14α demethylase; *ALOX5*: Arachidonate 5- lipoxygenase; *FDXR*: Adrenodoxin reductase; *FMO5*: Dimethylaniline monooxygenase [N-oxide-forming] 5; *KMO*: Kynurenine 3-monooxygenase; *MAOB*: Monoamine oxidase B; *PAH*: Phenylalanine hydroxylase; *POR*: Cytochrome P450 Oxidoreductase; *SQLE*: Squalene Epoxidase; *TBXAS1*: Thromboxane-A synthase 1).
